# Anthropometrically determined nutritional status of urban primary schoolchildren in Makurdi, Nigeria

**DOI:** 10.1186/1471-2458-11-769

**Published:** 2011-10-06

**Authors:** Daniel T Goon, Abel L Toriola, Brandon S Shaw, Lateef O Amusa, Makama A Monyeki, Oluwadare Akinyemi, Olubola A Alabi

**Affiliations:** 1 Department of Sport, Rehabilitation and Dental Sciences, Tshwane University of Technology, Pretoria, South Africa; 2 Centre for Biokinetics, Recreation and Sport Science, University of Venda, Thohoyandou, South Africa; 3 School of Biokinetics and Sport Science, North-West University, Potchestroom, South Africa; 4 Department of Statistics, University of Venda, Thohoyandou, South Africa; 5 Department of Food Science and Technology, The Polytechnic, Saki Campus, Saki, Oyo, Nigeria

## Abstract

**Background:**

No information exists on the nutritional status of primary school children residing in Makurdi, Nigeria. It is envisaged that the data could serve as baseline data for future studies, as well as inform public health policy. The aim of this study was to assess the prevalence of malnutrition among urban school children in Makurdi, Nigeria.

**Methods:**

Height and weight of 2015 (979 boys and 1036 girls), aged 9-12 years, attending public primary school in Makurdi were measured and the body mass index (BMI) calculated. Anthropometric indices of weight-for-age (WA) and height-for-age (HA) were used to estimate the children's nutritional status. The BMI thinness classification was also calculated.

**Results:**

Underweight (WAZ < -2) and stunting (HAZ < -2) occurred in 43.4% and 52.7%, respectively. WAZ and HAZ mean scores of the children were -0.91(SD = 0.43) and -0.83 (SD = 0.54), respectively. Boys were more underweight (48.8%) than girls (38.5%), and the difference was statistically significant (p = 0.024; p < 0.05). Conversely, girls tend to be more stunted (56.8%) compared to boys (48.4%) (p = 0.004; p < 0.05). Normal WAZ and HAZ occurred in 54.6% and 44.2% of the children, respectively. Using the 2007 World Health Organisation BMI thinness classification, majority of the children exhibited Grade 1 thinness (77.3%), which was predominant at all ages (9-12 years) in both boys and girls. Gender wise, 79.8% boys and 75.0% girls fall within the Grade I thinness category. Based on the WHO classification, severe malnutrition occurred in 31.3% of the children.

**Conclusions:**

There is severe malnutrition among the school children living in Makurdi. Most of the children are underweight, stunted and thinned. As such, providing community education on environmental sanitation and personal hygienic practices, proper child rearing, breast-feeding and weaning practices would possibly reverse the trends.

## Background

Anthropometry has become a practical tool for evaluating the nutritional status of populations, particularly of children in developing countries [1] and nutritional status is the best indicator of the global well-being of children [2]. One of the major global health problem faced by the developing countries, today is malnutrition [3,4]. Of course, Nigeria too, is not an exception to this problem of malnutrition [5,6]. The primary cause of ill-health and premature mortality among children, in developing countries is attributed to undernutrition [7]. In developing countries, it is postulated that poverty and ignorance are primary casual factors of malnutrition [6].

There are several studies [5,6,8-10], investigating the problem of undernutrition among children in different parts of Nigeria. However, scanty information exists regarding undernutrition among urban children in Makurdi, Nigeria. Furthermore, most previous studies are focused on children under five years, in neglect of pre-adolescent group. Also, while information on body fat [11], physical fitness [12,13], anthropometric profiles [14], body weight disorders [15], and centripetal fat patterning [16] exist on children in Makurdi, reliable data on their nutritional status are hardly available. Understanding the nutritional status of children has far-reaching implications for promoting the health of future generations [17]. Therefore, using anthropometric index, the present study evaluates the prevalence of undernutrition among pre-adolescent urban school children aged 9-12 years living in Makurdi, Nigeria. We thought that such data are essentially important from public health standpoint as they would provide reliable bases for instituting appropriate strategies to identify and combat factors associated with nutritional abnormalities in children.

## Methods

### Study area

This study was carried out in Makurdi, Benue State capital, which is located between latitudes 7° 13' North and 8° 00' North, and longitudes 8° 00' East and 9° 00' East. It occupies a total area of 77 379.32 square kilometers with an estimated population of 947 138 (1991 census) [18]. The town is situated 315 km southeast of Abuja, Nigeria's Federal Capital Territory.

The climate of Makurdi is characterised by tropical wet and dry seasons with an annual average temperature ranging from 21°C and 38°C, respectively. The annual average rainfall of this region is estimated at 1152.2 mm, of which approximately 88% falls in the months between April and October. The dry season normally starts in November and ends in March.

The Tivs are the predominant people in the area, although a good number of other ethnic groups such as Idomas, Igedes, Etulos and Jukuns also inhabit some parts of the area. The major language spoken in the area is Tiv although English is also widely spoken in schools, offices and markets.

Benue state is acclaimed as Nigeria's "food basket" because of its rich and diverse agricultural resources. In this regard, agriculture is the main industry in the area with about 85% of the labour force estimated to be wholly employed in it. Apart from the farming population, other people in the area are mainly employed or self-employed in civil service, petty trading and fishing.

The major staple foods are yam (*dioscorea*), rice and cassava (*manihot esculenta*). Fruits and vegetables are also grown and eaten in small quantity. Beef and fish are the main sources of protein.

### Sample

Data for this study were obtained from a cross-sectional survey designed to evaluate the physical fitness and body composition of children (aged 9-12 years) in Makurdi, Nigeria. Details regarding the methodology of the study have been reported elsewhere [14,15].

The study involved 19 schools randomly selected from a total of 38 schools within Makurdi metropolitan town. A two-stage probability sampling method was used. The first stage included the selection of schools and the second stage consisted of random sampling of boys and girls of each age category from the total enrolment in their schools. A representative sample of the schools from this area was drawn based on the official list obtained from the Benue State Universal Basic Education Board. Participating schools were randomly selected within the five geographical areas of Makurdi town (High-Level, Kanshio, North Bank, Wadata and Wurukum). Twenty schools were the targeted study sample. However, one school declined to participate in the study based on local administrative bureaucracy and was excluded. Participants were also randomly selected by balloting technique. In all selected schools children within the age 9 and 12 years were eligible to participate in the study and were measured. School records of birth were used to establish ages of participants in the study. Children with known health complications were excluded from the study. Out of 2139 participants, due to absenteeism and incomplete data of 124 participants, 2015 participants eventually completed the anthropometric measures in 2005. These measurements were collected from September to December by a team of eight trained research assistants. Written informed consent was obtained from the participants' parents or guardians and individual head teachers. Permission for the study was granted by the Benue State Universal Basic Education Board, Makurdi, Nigeria. The study also received approval from the Ethics Committee of Tshwane University of Technology, South Africa.

### Anthropometric measures

Height and weight were determined according to standard anthropometric methods (International Society for the Advancement of Kinanthropometry: ISAK) [19]. Height was measured to the nearest 0.1 centimeters (cm) in bare feet with participants standing upright against a mounted stadiometer. Weight was measured to the nearest 0.1 kilogramme (kg) with participants lightly dressed (underwear and T-shirt) using a portable digital scale (Tanita HD 309, Creative Health Products, MI, USA). BMI was computed as weight/height^2 ^(kg m^-2^).

### Nutritional status

In order to analyse the nutritional status of our sample, the weight and height of the boys and girls were compared to those of same aged boys and girls measured in the National Health and Nutritional Examination Survey in the USA (NHANES) [20]. The z-scores < - 2.0 was used to classified stunted and underweight children based on their HAZ and WAZ values. The WHO [21] classification for assessing severity of malnutrition by percentage prevalence ranges of these two indicators among children were followed [22]. Also, the BMI thinness classifications were calculated [23].

### Statistical analysis

Means and standard deviations were calculated for body mass, stature and BMI across sex and age groups. Differences in the mean body mass, stature and BMI were evaluated for boys and girls according to age-group, using an independent samples *t*-test. The z-scores of < -2.0 was calculated to derive HAZ and WAZ category of stunting and underweight, respectively. The children were classified on the basis of WHO 2007 BMI thinness classification [23]. Children with BMI of 17.0-18.5, 16.0-17.0 and < 16.00 were classified as grade 1, 2 and 3 thinness, respectively. Children with a BMI of 18.5-25.0, 25.0- < 30 and > 30 were categorized as normal, overweight and obesity, respectively. All statistical analyses were performed using Statistical Package for Social Sciences (SPSS) version 17.0. A probability level of ≤ 0.05 was considered to be statistically significant.

## Results

The anthropometric characteristics of the Nigerian children are indicated in Table 1. Overall, the mean values for body mass (Boys: 29.8 ± 4.4 kg; Girls: 31.5 ± 6.1 kg), stature (Boys: 137.2 ± 7.7 cm; Girls: 138.9 ± 8.1 cm) and BMI (Boys: 15.7 ± 1.8 kg.m^-2^; Girls: 15.7 ± 1.8 kg.m^-2^), were significantly higher in the girls compared to boys (p ≤ 0.05). Although there was no significant sex difference in the mean values of body mass among the 9 year-olds (p > 0.05), substantial differences were noted in those aged 11 to 12 years old (p ≤ 0.0001). The children in this study had mean stature ranging from 129.9 ± 5.3 cm to 142.1 ± 6.7 cm (boys) and 131.4 cm ± 5.8 to 145.4 cm ± 6.6 (girls). The girls were consistently taller than the boys in all age categories. Stature was found to increase with age regardless of sex.

**Table 1:** Characteristics of the participants

			Weight (kg)			Height (cm)			BMI (kg.m^-2^)		
Age	Boys n	Girls n	Boys Mean (SD)	Girls Mean (SD)	p-value	Boys Mean (SD)	Girls Mean (SD)	p-value	Boys Mean (SD)	Girls Mean (SD)	p-value
9	192	213	26.1 (2.8)	26.3 (3.4)	0.4632	129.9 (5.3)	131.4 (5.8)	0.0007*	15. 4 (1.3)	15.2 (1.6)	0.1503
10	279	303	28.9 (3.4)	29.7 (4.8)	0.0188*	135.0 (6.0)	136.1 (6.1)	0.0379*	15.8 (1.7)	16.0 (2.2)	0.2867
11	234	244	30.4 (4.0)	32.8 (4.4)	< .0001*	139.8 (6.1)	141.7 (5.7)	0.0004*	15.6 (1.9)	16.3 (1.9)	0.0001*
12	274	276	32.3 (4.3)	35.9 (6.2)	< .0001*	142.1 (6.7)	145.4 (6.6)	< .0001*	16.0 (2.1)	16.9 (2.6)	0.0001*

*Statistically significant (p ≤ 0.05); kg: kilograms; kg.m^-2^: kilograms per square meter; SD = standard deviations; n = number of participants

There was no significant sex difference in BMI at ages 9 and 10 years (p > 0.05). However, the mean difference was significant at ages 11 and 12 years, where girls exhibited significantly (p ≤ 0.0001) higher mean BMI values compared to the boys. In this study, BMI increased linearly in girls, while in boys a contrasting downward trend in BMI was observed at ages 10 to 11, which again increased at 12 years of age (Table 1).

Table 2 presents the gender-and age-specific means and standard deviations (SD) of weight-for-age z-score (WAZ) and height-for-age z-score (HAZ) values. The mean Z-scores of weight-for-age and height-for-age in children were -0.91(SD = 0.43) and -0.83 (SD = 0.54), respectively. No significant differences were noted in HAZ between boys and girls.

**Table 2:** Anthropometric indices of nutritional status in Nigerian children, by sex and age

		Height-for-age z-score		Weight-for-age z-score	
		
Age (years)	n	Mean	SD	Mean	SD
**Boys**					
9	192	-0.01	0.91	-0.81	0.45
10	279	-0.84	0.88	-0.83	0.44
11	234	-0.89	0.84	-0.93	0.41
12	274	-1.25	0.86	-1.07	0.40
Total	979	-1.0	0.89	-0.91	0.43
**Girls**					
9	213	-0.60	0.81	-0.75	0.46
10	303	-0.73	0.83	-0.71	0.58
11	244	-0.77	0.70	-0.81	0.41
12	276	-1.27	0.92	-1.04	0.58
Total	1036	-0.86	0.86	-0.83	0.54

*Statistically significant at (p < 0.05); n = number of participants; SD = standard deviation

The largest percentage of the children were moderately stunted (1062; 52.7%) HAZ and (1136; 56.4%) WAZ. Normal WAZ and HAZ occurred in 54.6% and 44.2% of the children, respectively (Table 3).

**Table 3:** Nutritional status of the participants using the Height-for-age Z-scores (HAZ) and Weight-for-Age Z-scores (WAZ)

	HAZ	WAZ
	
Nutritional status	n (%)	n (%)
Severe malnutrition	28(1.4)	-
Moderate (stunting)	1062(52.7)	874(43.4)
Normal (non stunting)	891(44.2)	1136(56.4)
Total	2015 (100)	2015 (100)

WAZ = Weight-for-age Z-score; HAZ = Height-for-age Z-score; % = percentage

**Figure 1:**
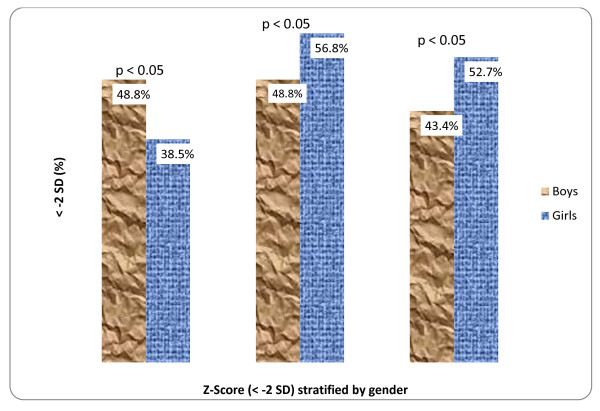
**Nutritional status of pre-adolescents school children stratified by gender**.

Shown in Figure 1 is the nutritional status of pre-adolescent school children stratified by gender. The prevalence of underweight status was higher in boys than the girls, and the difference was statistically significant (p = 0.004; p < 0.05). Conversely, girls tended to be more stunted compared to boys and the difference was statistically significant (p = 0.004; p < 0.05). Based on the WHO classification of severity of malnutrition, the prevalence of underweight and stunting was 31.3% among the children.

The results of BMI thinness classification of the participants according to gender and age groups (Table 4), indicates that age variations exist in the different BMI thinness classifications. Majority of the children exhibited Grade 1 thinness (77.3%), which was predominant at all ages (9-12 years) in both boys and girls. Gender wise, 79.8% boys and 75.0% girls fall within the Grade I thinness category. Only few boys (8.5%) and girls (15.8%) were in the normal group. Grade 2 thinness was 6.9% and 5.8% in boys and girls, respectively. Similarly, 4.6% of the boys and 3.0% of girls fall within the Grade 3 thinness category. Overweight and obesity was absent in boys at ages 9, 10 and 11 and in girls at ages 9 and 10 years.

**Table 4:** BMI thinness classification of pre-adolescent school children according to gender

Age (years)	BMI thinness classification*					
	
	< 16.00 Grade 3 thinness	16.0-17.0 Grade 2 thinness	17.0-18.5 Grade 1 thinness	18.5-25.0 Normal	25.0- < 30 Overweight	> 30 Obesity
	
	n (%)	n (%)	n (%)	n (%)	n (%)	n (%)
**Boys **(n = 979)						
9	3 (1.6)	14 (7.3)	167 (87.0)	8 (4.2)	0 (0)	0 (0)
10	8 (2.9)	13 (4.7)	229 (82.1)	29 (10.4)	0 (0)	0 (0)
11	15 (6.4)	24 (10.3)	176 (75.2)	19 (8.1)	0 (0)	0 (0)
12	19 (6.9)	17 (6.2)	209 (76.3)	27 (9.9)	1 (0.4)	1 (0.4)
Total	45 (4.6)	68 (6.9)	781 (79.8)	83 (8.5)	1 (0.1)	1 (0.1)
**Girls **(n = 1036)						
9	6 (2.8)	17 (8.0)	179 (84.0)	11 (5.2)	0 (0)	0 (0)
10	10 (3.3)	9 (3.0)	248 (81.8)	34 (11.2)	1 (0.3)	1 (0.3)
11	6 (19.4)	10 (4.1)	192 (78.7)	36 (14.8)	0 (0)	0 (0)
12	9 (3.3)	24 (8.7)	156 (57.2)	83 (30.1)	1 (0.4)	1 (0.4)
Total	31 (3.0)	60 (5.8)	777 (75.0)	164 (15.8)	2 (0.2)	2 (0.4)
Boys + Girls (n = 2015)	76 (3.8)	128 (6.4)	1558 (77.3)	247 (12.3)	3 (0.1)	3 (0.1)

*WHO 2007 BMI thinness classification

## Discussion

The study provides anthropometric data on the nutritional status in a group of preadolescent school children in Makurdi, Nigeria. Nutritional status is an integral component of the overall health of an individual [24], and provides an indicator of the well-being of children [25] living in a particular region. In this regard, the importance of the nutritional status of children in the developing countries should be emphasized, not only for the improvement of health of children in the coming generation, but also for the overall development of the concerned region in near future [26]. The findings of this present study reflect a seemingly high prevalence of malnutrition (31.3%) among the school children living in Makurdi. Table 5 compares the results of the nutritional status of the children in the present study with the results of other similar studies elsewhere. In this study, the prevalence of undernutrition, particularly stunting is higher than underweight.

**Table 5:** Nutritional status of children in the literature compared with the results of the present study

			Nutritional status			
				
Country	Sample	Age (years)	Stunting (%)	Wasting (%)	Underweight	Reference
Nigeria	331 boys and girls (pastoral, Southwest)	6-15	38.7	13.6	38.7	[9]
India	1206 boys and girls (West Bengal)	1-12	48.4 (F) 51.7 (M)	-	51.3 (F) 60.4 (M)	[22]
India	596 (Kerala), 1033(West Bengal), 2357 (Bihar) boys and girls	0-30	54† 39†† 23†††	-		[24]
India	353 boys and girls (Garhwal Himalayas)	0-12	78.5	80.1		[25]
India	1022 boys and girls (Madhya Pradesh)	0-5	51.6%	32.9%	61.6	[26]
Kenya	1383 boys and girls (rural Suba district)	5-17	15.8 (F) 27.5 (M)	6.3 (F) 10.6 (M)		[27]
Botswana		0-3	38.7	5.5	15.6	[28]
Democratic Republic of Congo (DRC)	8992 boys and girls	0-5	41.7 (F) 46.1 (M)	-	-	[29]
Egypt	143 boys and girls (Peri-urban, Alexandria	< 3	19	3	7	[30]
Malaysia	399 boys and girls (Kuala Lumpur)	6-8	47	36		[31]
Mexico			28.8	40.9		[32]
South Africa	671 boys and girls (rural Agincourt)	12-59	18	7	10	[33]
South Africa	296 boys and girls (rural Lebowa)	6-14	54 (F) 60 (M)	6 (F) 8 (M)	38 (F) 62 (M)	[34]
Uganda	1003 boys and girls (Kumi district)	9-15	2.6 (F) 3.9 (M)	-	5.6 (F) 7.6 (M)	[35]
Nigeria	2015 boys and girls (Urban, Makurdi)	9-12	52.7	-	43.4	Present study

% = percentage; † = Bihar; †† = West Bengal; ††† = Kerala; F = females; M = males

Stunting and underweight are comparatively higher among the Indian samples [24-26] than the children in our present study. Of course, India has been reported to have had the highest prevalence of childhood malnutrition in the world [36]. Apart from studies involving Indian children, our sample had far higher percentage of undernutrition compared to other children in other countries.

Consistent with other previous studies in sub-Saharan Africa [27,37-44], and elsewhere [26,45], our study showed stunting to be higher in male than in female children. Contrastingly, in Chowdburg et al. [46] study, the prevalence of stunting (21.7%) and wasting (35.8%) was higher in girls compared to boys (13.8% stunting and 22.7% wasting). However, no sex difference was reported in any of the three states (Bihar, West Bengal, Kerala) among Indian [24] children. Collectively, stunting occurred in the 52.7% of the children, a figure which is higher compared to studies conducted on Nigerian children in other regions of the country. For example, Abidoye et al. [5] study reported 34.5% stunted children among 1-4 year old children in urban ghetto in Lagos. With an estimated population of more than 140 million [47], the prevalence of stunting in children under 5 years of age is 38.3% [48].

Underweight is used as a composite indicator to reflect both acute and chronic undernutrition, although it cannot distinguish between them [22]. The results of the present study indicated that 52.7% of the children are underweight, and boys are more underweight (48.8%) compared to girls (38.5%). The prevalence of underweight found in this study is high compared to [5,9,41] studies among Nigerian children. These studies reported the prevalence of underweight occurring more in boys than girls similar to our findings in this study. Also, similar results were reported from studies in other countries [49]. It is difficult to explain the variation in the level of underweight between boys and girls seen in this study. Whether both sexes are subjected to different conditions of nutrition and dietary intake is only speculative, as these were not assessed in the present study.

Using WHO 2007 BMI thinness classification, age variations exist in the different BMI thinness classifications (Table 4). While majority of the children exhibited Grade 1 thinness (77.3%), which was predominant at all ages (9-12 years) in both boys and girls, overweight and obesity was absent in boys at ages 9, 10 and 11 and in girls at ages 9 and 10 years. Generally, there is low prevalence of overweight and obesity in our sample when compared with children in developed countries. A possible explanation of the very low prevalence of overweight and obesity and high prevalence of stunted, underweight and thinned children in Makurdi, Nigeria, could be that unlike in most Western countries, the children have limited access to high-calorie snacks and fast food which are hardly affordable. Another plausible explanation is that the Nigerian children may have been engaged in more physically demanding activities compared to their contemporaries in other countries. Majority of the children walk to school daily regardless of the distance and this may have enhanced their physical activity levels [15]. Recent studies have reported higher levels of physical activity in children associated with active travel to school [50,51].

The high prevalence of both chronic and acute malnutrition observed in the present study is unexpected from an urban region. However, the fact is that most of the children attending primary school in this region are from relatively low socio-economic backgrounds. Therefore the low socio-economic background of these children suggests that factors such as education, occupation and economic status of parents may also account for the high prevalence of undernutrition among our cohort. Could the appalling nutritional situation among our sample be linked to poverty, then the efficacy of the Nigerian government's nutritional intervention programme becomes questionable. Programmes such as the School Meal Programme (SMP), targeted towards heads of households; the Family Support Programme (FSP); and Women Empowerment Programmes (WEP) are not sustainable over time, and some have limited effects [52]. Therefore, making these programmes viable and result-oriented would positively improve the children's nutritional status.

Poor water and sanitation is associated with increased frequency of water and sanitation related morbidity [53]. It is observed that in Makurdi town, the rate of water supply and other sanitary and hygienic facilities are poor. Access to safe water and sanitation is limited to majority of the population, especially those from poor socio-economic backgrounds. Only few people have residential pipe borne water. The majority of the people are served by irregular supply of water by public taps or street vendors. It is possible that most children in this study lived in houses with irregular supply of water. This has implications for the health of these children as the WHO recommended daily water requirements of 200-300 liters per person may be elusive. With poor access to safe water and sanitation, diarrhoeal diseases are more likely to be rampant and could lead to poor nutritional status.

The major staple foods of the people in this region are yam (*dioscorea*), rice and cassava (*manihot esculenta*). These food items are mainly carbohydrates. It is possible that in view of the low socio-economic status of the children's parents, many cannot afford to buy meat or fast foods. Such scenario could impact on the children's nutritional status.

Several limitations should also be considered when interpreting the results of the present study. It should be noted that data was not collected on birth weight of the children that might be helpful in further understanding the genesis of the deviation of weight/height scores from the "standard population" observed in this present study, which might likely reflect previous states of malnutrition. Also, data on dietary intake was not available. Again, body mass index-for-age might not necessarily be appropriate in suggesting that so many of the children in this study are severely wasted, stunted and thinned. BMI is only a proxy measure of nutritional status, with an inability to differentiate between fat-free mass and the adipose tissue. Perhaps another pertinent issue concerns the definition of appropriate cut-off points for evaluating nutritional status. The cut-off points used to screen nutritional status in this study was derived based on arbitrarily set BMI values, which reflect changes in BMI that occur with age in the boys and girls [15]. It could be observed that different methodologies were utilized for assessing the nutritional status, which seems justified on the basis that there is need to use international reference data to obtain information about population of developing countries and adequately insert the information into a pattern of general global changes and trends [27]. Besides, only schoolchildren in public schools were studied, so that the results of the present study do not necessarily apply to all Nigerian children living in Makurdi.

Despite these limitations, the study has provided important new information on nutritional status of Nigerian children in Makurdi region, which scanty information exist. Thus, from a public health standpoint, such data could be of paramount importance in formulating urgent intervention strategies in order to stem the health hazards of malnutrition among the Nigerian children in this region.

## Conclusion

There is severe malnutrition among the school children living in Makurdi, Nigeria, as most of the children are underweight, stunted and thinned. As the quality of future human resources depends on the present day children, improvement of the nutritional level of today's children should be given top priority [24]. Perhaps, education concerning environmental sanitation and personal hygienic practices, proper child rearing, breast-feeding and weaning practices tailored to the community in this region would possibly reverse the trends.

## Competing interests

The authors declare that they have no competing interests.

## Authors' contributions

DTG was the primary investigator for the study, designed the study, supervised data collection and wrote the paper. ALT advised on data collection and helped write the paper. BSS advised on the design of the study and wrote the initial draft manuscript. LOA, MMA AO participated in the analysis of the findings and writing the paper. OAA participated in the fieldwork and data collection. All authors participated in review of the manuscript, read and approved the final manuscript.

## Pre-publication history

The pre-publication history for this paper can be accessed here:

http://www.biomedcentral.com/1471-2458/11/769/prepub
